# Pathway Switching Explains the Sharp Response Characteristic of Hypoxia Response Network

**DOI:** 10.1371/journal.pcbi.0030171

**Published:** 2007-08-31

**Authors:** Yihai Yu, Guanyu Wang, Rahul Simha, Weiqun Peng, Frank Turano, Chen Zeng

**Affiliations:** 1 Department of Physics, George Washington University, Washington, D.C., United States of America; 2 Department of Computer Sciences, George Washington University, Washington, D.C., United States of America; 3 Department of Biological Sciences, George Washington University, Washington, D.C., United States of America; Lawrence Berkeley National Laboratory, United States of America

## Abstract

Hypoxia induces the expression of genes that alter metabolism through the hypoxia-inducible factor (HIF). A theoretical model based on differential equations of the hypoxia response network has been previously proposed in which a sharp response to changes in oxygen concentration was observed but not quantitatively explained. That model consisted of reactions involving 23 molecular species among which the concentrations of HIF and oxygen were linked through a complex set of reactions. In this paper, we analyze this previous model using a combination of mathematical tools to draw out the key components of the network and explain quantitatively how they contribute to the sharp oxygen response. We find that the switch-like behavior is due to pathway-switching wherein HIF degrades rapidly under normoxia in one pathway, while the other pathway accumulates HIF to trigger downstream genes under hypoxia. The analytic technique is potentially useful in studying larger biomedical networks.

## Author Summary

A complex biomolecular network utilizes different pathways to perform different functions. However, the interactions within the network are typically so complicated that the pathway structure is usually hidden. By some mathematical techniques, the pathways can be identified and possibly decoupled, whereby the insightful details of the network can be exposed. As an example, we study in this paper the hypoxia response network that manifests a dramatic switch-like behavior for certain sets of rate constants: a slight change of the oxygen concentration close to a critical value will lead to distinct reaction patterns. By a technique called extreme pathway analysis, the network is decoupled into three major and some minor pathways. Flux distribution among these pathways can thus be measured by integrating the ordinary differential equations for any given set of rate constants. For the sets of rate constants where the switch-like behavior is observed, we found that such a behavior is due to the switching of flux between two of the three major pathways.

## Introduction

**Figure 1:**
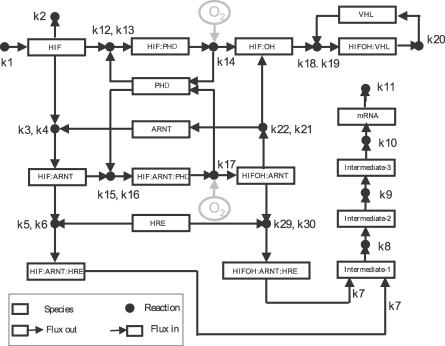
Diagram of the Assumed Core Subsystem of the Hypoxia Response Network Redrawn on the Basis of [1] For simplicity we use HIF to represent HIF*α*.

Molecular oxygen is the terminal electron acceptor in the mitochondrial electron transport chain. Hypoxia, or oxygen deficiency, induces a number of metabolic changes with rapid and profound consequences on cell physiology. A hypoxia-induced shortage of energy alters gene expression, energy consumption, and cellular metabolism to allow for continued energy generation despite diminished oxygen availability. A molecular interaction map of the hypoxia response network has been proposed [1–3] on the basis of analyzing conserved components between nematodes and mammals. The key element in this network, hypoxia-inducible factor (HIF), is a master regulator of oxygen-sensitive gene expression [4–6]. HIF is a heterodimeric transcription factor which consists of one of the three different members (HIF-1*α*, HIF-2*α*, and HIF-3*α*) and a common constitutive ARNT subunit which is also known as HIF*β*. The system also includes an enzyme family: prolyl hydroxylases (PHDs), which directly sense the level of oxygen and hydroxylate HIF*α* by covalently modifying the HIF*α* subunits. It is very likely that reactive oxidative species (ROS), which are a byproduct of mitochondrial respiration, are also involved in oxygen sensing by neutralizing a necessary cofactor, Fe^2+^, for the hydroxylation of HIF*α* by a PHD [7–10]. There are three members in this enzyme family: PHD1, PHD2, and PHD3. The hydroxylated HIF*α* is then targeted by the von Hippel-Lindau tumor-suppressor protein (VHL) for the ubiquitination-dependent degradation. Hypoxia response element (HRE) is the promoter of the hypoxia-regulated genes, and the occupancy of HRE controls the expression levels of these genes. The cascade in Figure 1 (reproduced from Figure 2 of [1]) consists of an input (the concentration of oxygen) and an output (the activation of promoters that are under control of HREs) as the core network. The network is characterized by a switch-like behavior, namely the sharp increase of HIF*α* when the oxygen decreases below a critical value, followed by a sharp increase of HRE occupancy. It was observed experimentally on many cell lines including Hela cells [11] and Hep 3B cells [12] that HIFα increases exponentially as the oxygen concentration decreases.

**Figure 2:**
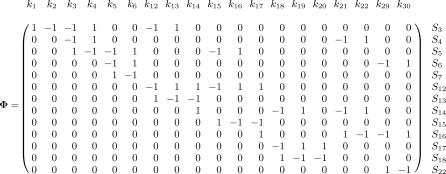
Stoichiometric Matrix of the Reduced Hypoxia Response Network The rows represent the 13 variable molecular species in Table 1, and the columns represent the 19 reactions in Table 2.

**Table 1:**
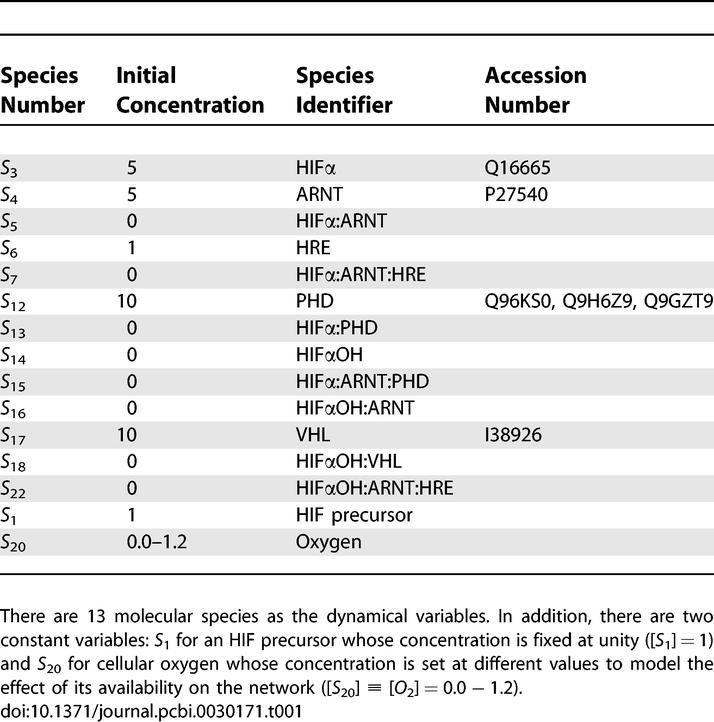
Molecular Species of the Reduced Network

**Table 2:**
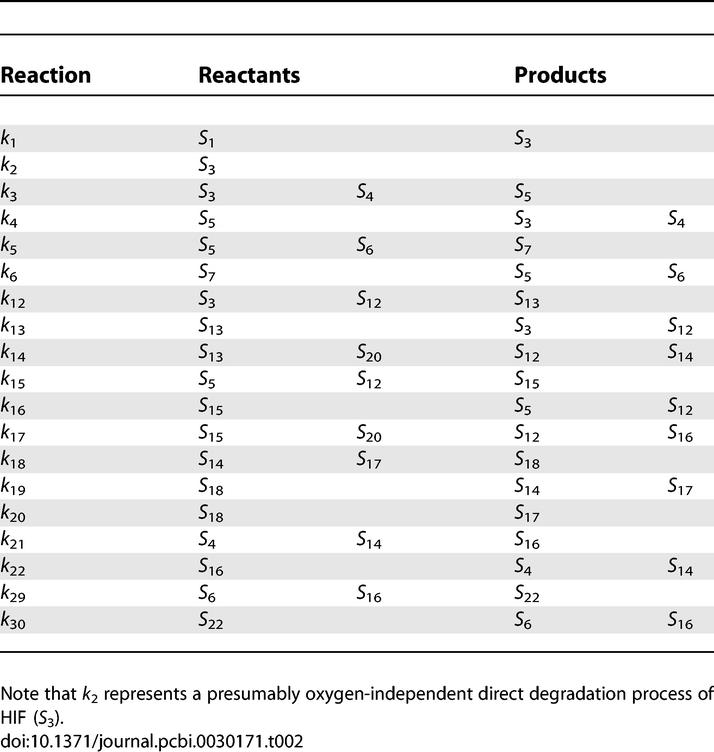
19 Reactions of the Reduced Network and Corresponding Reactants and Products

The past two decades have seen a growing body of work on the use of mathematical modeling to help uncover both general principles behind molecular networks and to provide quantitative explanations of particular network phenomena [13] that may one day have sufficient predictive power to accurately model large subnetworks of the cell. In this sense, Kohn et al. [1] have successfully modeled the switch-like response characteristics of HRE occupancy, by numerically integrating a system of ordinary differential equations (ODEs) involving a score of molecular species related to hypoxia. The large model, however, does not identify the smaller components that are actually responsible for the switch-like response and that may occur in other such networks. Furthermore, a numerical solution does not provide the type of insight that mathematical formulas can. At the same time, it is virtually impossible to solve symbolically the type of nonlinear differential equations that model reactions. In this context, methods are desirable that are both tractable, that reduce a system to its key components, and that are not solely reliant on numerical solution.

Extreme pathway analysis (EPA) is one such recently developed method [14–16]. In this method, the dynamics of interactions between species are formulated as a Boolean network in which the state of a gene is represented as either transcribed or not transcribed. Upregulation and downregulation of genes are captured through an appropriate sign (plus or minus) and a scaling constant. The Boolean network is then formulated as a matrix of interaction rules that is then analyzed to help reveal key components and their contributions to the dynamic behavior [16]. The theory of matrices then allows us to look for vectors that characterize the matrix in ways that are helpful for further analysis. The EPA technique, in particular, finds vectors (extreme pathways) that correspond to the boundaries of the space of steady-state solutions to the differential equations. We note that similar methods, such as flux balance analysis (FBA) and elementary modes analysis, have been developed in other contexts [17–19]. They essentially yield the same results [18], which have been verified by ExPa [20] and CellNetAnalyzer [21,22]. These methods provide a way out of the intractable complexity of sizable molecular networks [23–26].

Our contribution is to go beyond this type of matrix approach and provide a detailed quantitative analysis that explains the observed behavior in the models. This is achieved by combining elementary pathway identification via EPA, which depends solely on the network topology, and the detailed analytical as well as numerical analysis of the governing differential equations in the model, which allows studies of the phase space spanned by the mostly unknown rate constants in the differential equations. Specifically, EPA is first used in our approach to decompose the original network into several underlying pathways. Following this, we make some reasonable approximations to facilitate analytic solution. We show that this analytic solution, in the case of the hypoxia network, explains the switch-like behavior. This explanation is confirmed by comparing the numerical output of the simplified model with the numerical output of the complete (and complex) differential equation model.

A second contribution of this paper is to highlight a particular mechanism of pathway-switching or pathway branching effect [27] that appears to cause the sharp response to oxygen concentration. In particular, we examine the flux redistribution among the elementary pathways as a function of oxygen concentration. We also identify the key molecular species involved in the subcomponent of the network and show quantitatively how the response of this subcomponent exactly matches the overall response and thus is responsible for it. For hypoxia, our analysis suggests that the cycle of abundant production and efficient degradation of HIFα plays the main role in the sharp response.

## Results

### Model Description

For consistency and ease of understanding, we use the notation and nomenclature in [1] and use their 23-species network and differential equation model as the starting point. With this background, the original network shown in Figure 2 of [1] can be further reduced in the following way. Kohn et al. [1] have shown that the feedback of mRNA is not necessary for the switch-like behavior. We therefore eliminate this feedback loop (reaction *k*_32_). Hence, Transcript intermediate 1, 2, and 3 (Species 8, 9, and 10) can also be dropped, as well as the associated reactions: *k*_7_, *k*_8_, *k*_9_, *k*_10_, and *k*_11_, because they do not affect the dynamics of the network. Species 23 is only the joint name of HIF*α*:ARNT:HRE (Species 7) and HIF*α*OH:ARNT:HRE (Species 22); therefore, it is dropped. HIF*α* precursor (Species 1) is a constant and is thus dropped, because its information can be simply encoded in the reaction *k*_1_. The degradation products (Species 2) are also eliminated because they are assumed to leave the network immediately after their production and do not affect the dynamics. Similarly, species 19, 20, and 21 do not contribute to the dynamics and are therefore removed. The resulting network is summarized in Tables 1 and 2, where there are 13 molecular species and 19 reactions in total. The system can be described by the following set of ODEs where [*S_n_*] stands for the concentration of species *n* as tabulated in Table 1 and [*O*_2_] indicates the input cellular oxygen concentration. Table 2 shows the specific reaction each rate constants *k_n_* represents. The real values of *k_n_* are from [1]. Note that the ODE system below is typical: the terms are based on mass-action principles and, taken together, result in complex behavior not readily discernible by examining the equations. We also dropped the precursor concentration [*S*_1_] since it is set to unity.

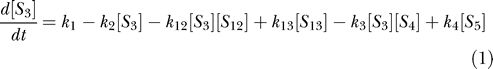


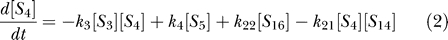


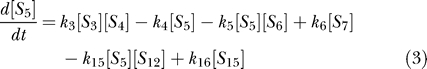


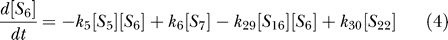


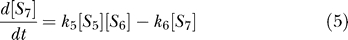


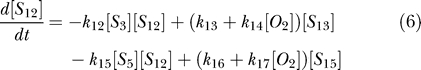


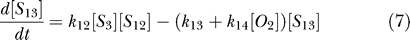


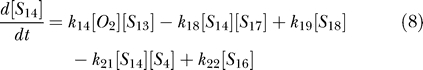


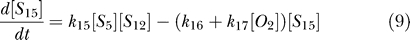


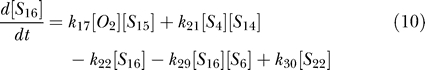


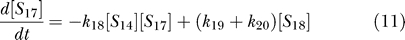


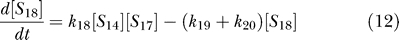


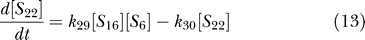



### Network Decomposition by Extreme Pathway Analysis

**Figure 3:**
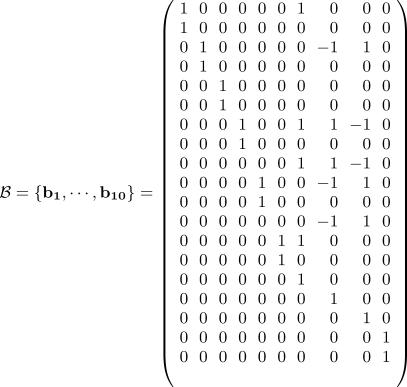
Set of Theoretically Feasible Basis Vectors **B**

**Figure 4:**
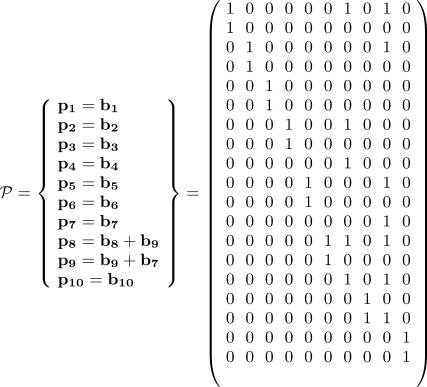
Set of Both Theoretically and Biochemically Feasible Basis Vectors **P**

This section assumes some familiarity with linear algebra. The 13 × 19 stoichiometric matrix Φ of the reduced hypoxia response network (Tables 1 and 2) is shown in Figure 2 (for details, see Materials and Methods). The rank of Φ is computed and shown to be 9, indicating that there are only nine independent molecular species to serve as constraints for the analysis. Therefore, the dimension of the corresponding null space is 10. The linearly independent basis *B* vectors are generated by Matlab 6.5 and are shown in Figure 3. According to the constraint that no negative values are allowed in the basis vectors, we can uniquely transform basis *B* into basis *P* as shown in Figure 4. Both **b**_8_ and **b**_9_ have negative terms. **b**_8_ has to be transformed first, otherwise there will be no +1 to cancel out the −1 at the twelfth row of **b**_8_. Each −1 in **b**_8_ is canceled out through the operation **b**_8_ + **b**_9_. In the second step, one has to use **b**_7_ to cancel −1 at the ninth row of **b**_9_. In this way, we obtain the set of basis vectors *P*. The above analysis indicates that the dimension of this null space is the same as the number of edges for its corresponding convex cone [28], which is the algebraic basis for extreme pathways [14] and elementary modes [29].

**Figure 5:**
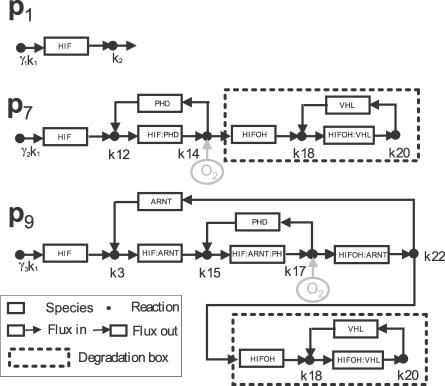
Graphical Representations of the Three Underlying Pathways of HIF*α* Degradation: **p**_1_, **p**_7_, and **p**_9_ Through **p**_1_, HIF*α* is degraded directly by *k*_2_. In **p**_7_, HIF*α* first binds with PHD after synthesis. HIF*α*:PHD is then dissociated into PHD and HIF*α*OH with the participation of oxygen. HIF*α*OH then binds with VHL to form HIF*α*OH:VHL. Finally, the dissociation of HIF*α*OH:VHL concludes HIF*α* degradation. The pathway **p**_9_ differs from **p**_7_ only in that HIF*α* first binds with ARNT after synthesization.

**Figure 6:**
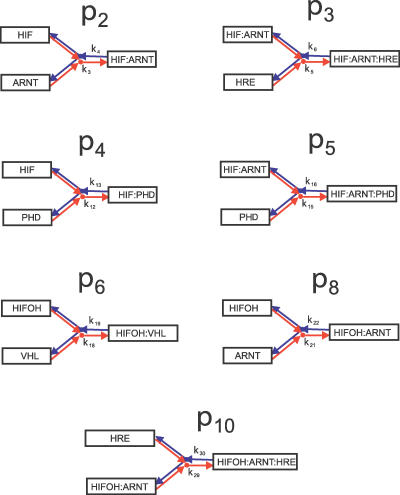
Simple Association and Dissociation pathways **p**_2_, **p**_3_, **p**_4_, **p**_5_, **p**_6_, **p**_8_, and **p**_10_ The associations (dissociations) are illustrated in red (blue).

The ten basis vectors of *P* represent ten underlying pathways of the hypoxia network. They are illustrated in Figures 5 and 6, from which one finds two distinct patterns: **p**_1_, **p**_7_, and **p**_9_ belong to the HIF*α* degradation pathways (Figure 5) and the others belong to the simple association–dissociation pathways (Figure 6). More specifically, through **p**_1_, HIF*α* is directly degraded by reaction *k*_2_, a presumably oxygen-independent degradation pathway; whereas in oxygen-dependent pathways **p**_7_ and **p**_9_, the hydroxylated HIF*α* is recognized by the VHL that channels it through a ubiquitin degradation component that is shown as the dotted box in Figure 5. Even though **p**_1_, **p**_7_, and **p**_9_ are all elementary modes [29], they can share certain reactions of the network. For example, the total influx for HIF*α* synthesis from a precursor can thus be decomposed into three parts with the overall rate constant *k*_1_ being given by *γ*_1_ *k*_1_, *γ*_2_ *k*_1_, and *γ*_3_ *k*_1_, where *γ*_1_ + *γ*_2_ + *γ*_3_ = 1.

The pathways **p**_7_, and **p**_9_ are almost the same. The only difference is that HIF*α* is associated with ARNT in the middle part of the pathway **p**_9_. Therefore, ARNT must be functionally very important, otherwise it would be hard to explain why the two underlying pathways, which should play significantly different roles, look so similar. Indeed, HIF*α* degrades differently through the two pathways. The k-sets were selected in [1] on the basis that they produced a switch-like behavior. For all the three k-sets, it was observed that HIF*α* has high affinity to PHD and low affinity to ARNT. The former is consistent with the usual case of high enzyme-substrate binding affinity. This implies that **p**_9_ is not the major degradation pathway because HIF*α* does not bind with ARNT very well. Moreover, **p**_9_ is immediately adjacent to HRE, which suggests its major role is to deliver signals to activate the promoters of hypoxia-regulated genes. As a signal transducer, the rate constant *γ*_3_ *k*_1_ itself need not to be high; what the downstream genes are sensitive to is *d*(*γ*_3_ *k*_1_)/*dt*. Therefore, we hypothesize that there is a negligible flux through **p**_9_ (or *γ*_3_ ≈ 0). To verify our hypothesis, we calculate the *γ*_1_, *γ*_2_, and *γ*_3_ values, as the indication of the relative importance of **p**_1_, **p**_7_, and **p**_9_ in HIF*α* degradation, at different oxygen levels. The results are given in Table 3. Note that [*O*_2_] = 0.1 and [*O*_2_] = 1.0 represent typical low and high oxygen levels according to [1]. One sees that the pathway **p**_9_ is always much less important than the other two as far as HIF*α* degradation is concerned. The majority of HIF*α* gets degraded via either **p**_1_ at low oxygen or **p**_7_ at high oxygen. The comparison of hypoxia response network and heat shock response network [30] as in Table 4 shows the similarity between these two networks with respect to the issue of affinity. The huge difference in the affinity can clearly separate the underlying pathways and assign different functions to them. This is also the basis for the Goldbeter-Koshland model [31]. We tested our method to all three parameter sets (k-sets 1, 2, and 3) in [1], and find that the analytical results are almost identical with those of the direct simulations of the entire network, which strongly validates our approximation. For the rest of the paper thereafter, we only report numerical results for k-set 1.

**Table 3:**
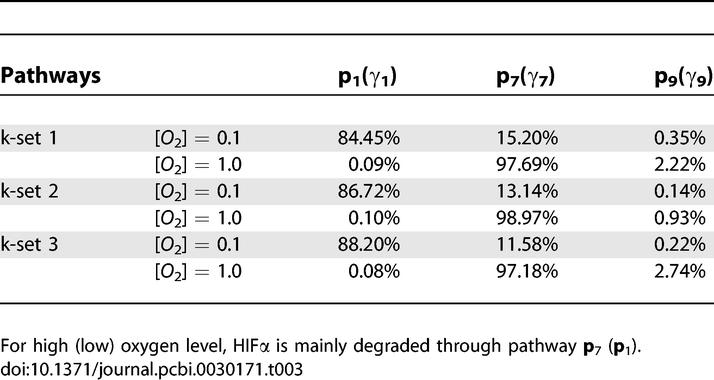
Flux through Pathways **p**_1_, **p**_7_, and **p**_9_ at [*O*_2_] = 0.1 and [*O*_2_] = 1.0 for the Three Sets of Parameters Given in [1]

**Table 4:**
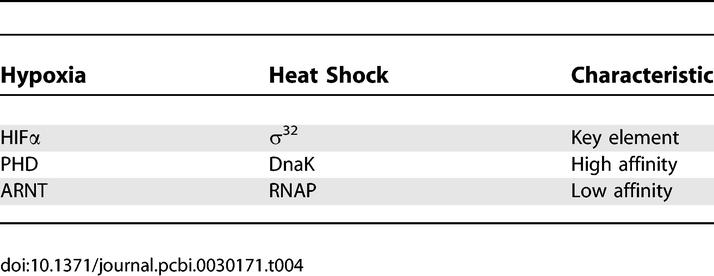
Comparison between Hypoxia Response Network and Heat Shock Response Network

### Analytical Solution to the Decomposed Hypoxia Response Network

The EPA method gives us a starting point from which to analyze our reaction network in greater and more revealing detail. The verification of our hypothesis implies that the pathways associated with **p**_9_ could be neglected in the first place. The following equations describe the combination **p**_1_,** p**_4_,** p**_6_, and** p**_7_, which constitute the oxygen sensing mechanism:

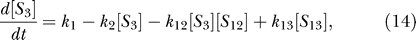


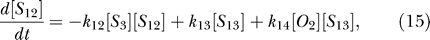


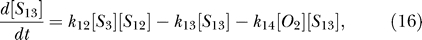


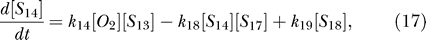


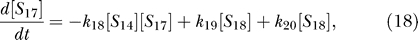


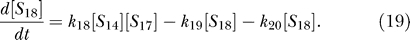



Note the differences between Equations 1, 6, and 8, and Equations 14, 15, and 17. The **p**_9_ related elements have been omitted due to their smallness. By setting the left-hand sides of the above equations to zero, one obtains the steady state equations:

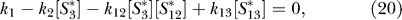


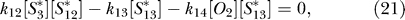


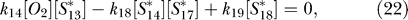


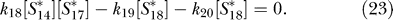



The total amount of PHD is conserved: PHD is either in the form of PHD (*S*_12_) or HIF*α*:PHD (*S*_13_). This implies

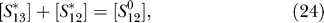
where 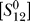 is the initial concentration of PHD. By some derivation, the following equation is obtained:

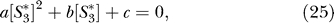
where *a* = *k*_2_ *k*_12_, *b* = *b*_1_ + *b*_2_[*O*_2_], *c* = c_1_ +*c*_2_[*O*_2_], where

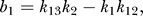


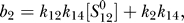


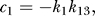


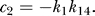



Since *c* < 0, Equation 25 has one and only one reasonable root

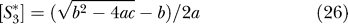



Note that none of the species and reactions in the degradation box is present in Equation 26, which indicates that the components in the degradation box are not responsible for the sharp response curve. Once 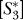 is determined, the analysis of the remaining network (**p**_2_, **p**_3_, **p**_5_, **p**_6_, **p**_8_, p_9_, and **p**_10_) becomes straightforward, and the results are given in the section “Additional results.” In fact, these results can be further simplified (see Equations 30 and 43 for 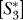 and 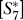 ).

**Figure 7:**
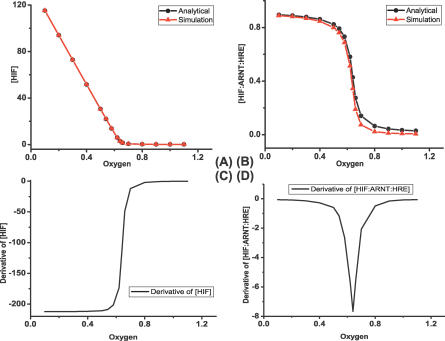
Steady State Values of [HIF*α*] and [HIF*α*:ARNT:HRE] (A) The comparison between analytical and numerical solutions of [HIF*α*]. (B) The comparison between analytical and numerical solutions of [HIF*α*:ARNT:HRE]. (C) The first derivative of [HIF*α*] with respect to [*O*_2_]. (D) The first derivative of [HIF*α*:ARNT:HRE] with respect to [*O*_2_].

Figure 7A and 7B shows the steady-state values of [HIF*α*] (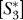 ) and [HIF*α*:ARNT:HRE] ( 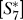 ) at different oxygen values. The red lines depict the simulation results obtained by the numerical integration of the ODE system (1–13) until the steady state is reached. The black lines depict the analytical solutions that are obtained by the algebraic Equations 26 and 56. To better determine the critical point of pathway switching, we calculate ∂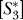 /∂[*O*_2_] and ∂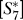 /∂[*O*_2_]. The results are shown in Figure 7C and 7D. One sees that both ∂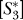 /∂[*O*_2_] and ∂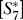 /∂[*O*_2_] change abruptly in a very narrow region of [*O*_2_], with the rest of the values almost zero. One observes that the critical point is about [*O*_2_]*^c^* = 0.65.

We thus show that the sharpness of the response curve can be determined analytically, instead of exhaustively enumerating [*O*_2_] values combined with time-consuming numerical integration of a large number of ODEs at each [*O*_2_] value.

## Discussion

EPA is a powerful, yet simple tool that can significantly reduce the complexity of the original network and thus make further analytical effort feasible. In this paper, the additional analysis explains precisely the sharp reaction to oxygen of the network as a whole. The clear separation of **p**_7_ and **p**_9_ indicates their different functions: the pathway **p**_7_ and its other associated pathways constitute the sensing of ambient molecular oxygen; in contrast, the pathway **p**_9_ and its associated other pathways are responsible for the signal transduction to form the promoters of hypoxia-regulated genes. Most importantly, the simplification allows for a complete explanation of the switch behavior and a clear presentation of the relations between 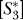 and [*O*_2_], 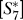 and [*O*_2_], 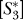 and 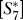 . The first step below explains the sharp HIF*α* stabilization. The second step explains the sharp HRE occupancy that is induced by the HIF*α* stabilization.

**HIF*α* stabilization**. This involves the dissociation of pathways **p**_1_, **p**_4_, **p**_6_, and **p**_7_ from the whole network, due to the fact that the flux through **p**_9_ is always small and can be neglected. It reveals a critical value that corresponds to the switching between pathways **p**_1_ and **p**_7_. Since an abrupt change often relates to the notion of singularity in mathematics, we proceed to see if a singularity can be found. Under nomoxia, 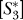 ≈ 0, and 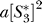 can be neglected in Equation 25, which yields

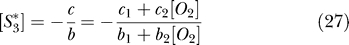



One immediately finds the singularity

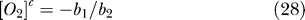



For k-set 1 in [1], one obtains [*O*_2_]*^c^* = 0.64, which is exactly the critical value found in Figure 7. When the oxygen level decreases to a value close to [*O*_2_]*^c^*, the denominator in Equation 27 becomes very small and *a* 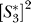 can no longer be ignored. Moreover, the term *c* in Equation 25 can be ignored compared with the large value of 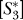 . One thus has

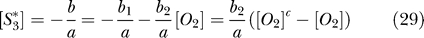



This explains the linear decrease of 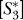 versus [*O*_2_] increasing in Figure 7A. In summary, one has

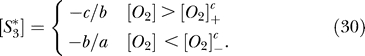



One can check that *k*_2_ can be ignored in the upper branch of Equation 30 due to its smallness, which again demonstrates that the pathway **p**_1_ is not important under nomoxia. The very smallness of *k*_2_ reflects the importance of **p**_1_ under hypoxia, for *k*_2_ exists at the denominator of the lower branch of Equation 30.

**HRE occupancy**. The remaining pathways reveal how HIF*α* stabilization triggers a sharp increase of HIF*α*:ARNT:HRE, namely the sigmoid curve of 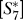 versus [*O*_2_]. We conclude that the magnitude of HIF*α* is crucial for the sharpness of the curve. To show this, we need Equations 47, 48, 53, and 54. By removing the terms that are negligible, these equations turn into

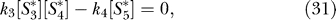


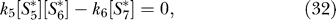


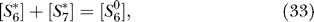


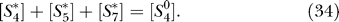



Equation 31 holds because *k*_15_ 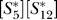 and *k*_16_ 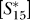 are far less than *k*_3_ 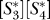 and *k*_4_ 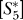 . Equation 33 holds because 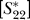 is far less than 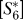 and 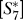 . Equation 34 holds because 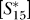 , 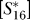 , and 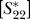 are far less than 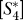 , 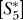 , and 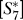 . The validity of the above approximations can be easily checked. For example, for [*O*_2_] = 0.1, one finds *k*_3_ 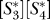 = *k*_4_ 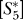 = 1.66, *k*_15_ 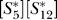 = *k*_16_ 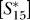 = 0.005, 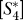 = 1.19, 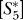 = 2.69, 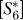 = 0.11, 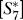 = 0.89, 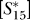 = 0.23, 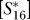 = 0.0016, and 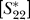 = 0.0005. From Equations 31–34, one obtains

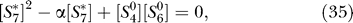
where α = α_1_ + α_2_/ 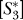 , α_1_ = 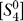 + 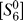 + *k*_6_/*k*_5_, and α_2_ = *k*_4_ *k*_6_/(*k*_3_ *k*_5_). 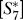 has one and only one reasonable solution

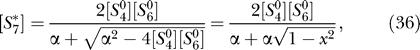
where *x* = 2 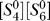 /α. Taylor expanding 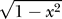 , yields

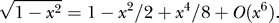



No matter what 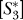 value is, x < 2 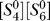 /α_1_ = 0.74, so 1 − x^2^/2 > 0.73 and *x* ^4^/8 < 0.0375. Therefore 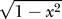 ≈1 − *x* ^2^/2 and

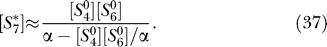



Under nomoxia 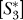 is small, so α ≈ α_2_/ 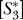 and 1/α *≈* 0. From Equation 37 one has

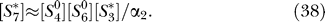
which is also small. Under hypoxia, 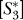 is large, and thus

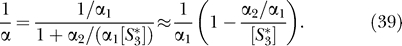



By substituting Equation 39 into Equation 37, one obtains the important relation between 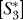 and 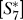 :

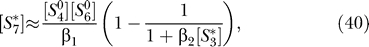
where

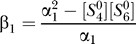
and

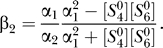



By substituting Equation 29 into Equation 40, one obtains

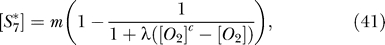
where *m* = 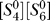 /β_1_ and λ = β_2_ *b*_2_/*a*. It is well-known that Equation 41 represents a sigmoid curve with *m* controlling the saturation value and λ controlling the sharpness. By ignoring the small term *k*_6_/*k*_5_ in the expression of α_1_, one finds *m* is a function of 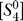 and 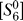 only. One also finds

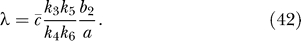



Here

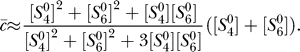



The association (disassociation) constants *k*_3_, *k*_5_ (*k*_4_, *k*_6_) exist in the numerator (denominator) of the term *k*_3_,*k*_5_/(*k*_4_,*k*_6_), which implies that the higher the affinity, the sharper the response. The third term *b*_2_/*a* is proportional to the HIF level. In summary, our analysis yields

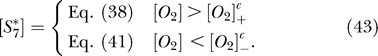



**Figure 8:**
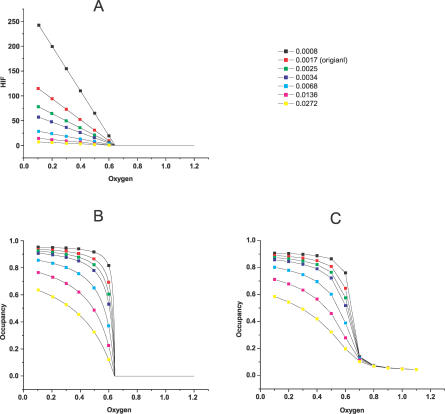
Different HIF and HRE Occupancy Responses at Different *k*_2_ Values (A) is obtained by the analytical solution Equation 29; (B) is obtained by the analytical solution Equation 41; (C) is obtained by the numerical integration of Equations 1–13; (A) and (B) reveal how HIF*α* controls HRE occupancy: the larger the HIF*α* level is, the sharper the HRE occupancy response. The validity of our analytical method is demonstrated by the close resemblance of (B) and (C).

One sees that HIF*α* is the key to triggering the HIF*α*:ARNT:HRE response. As long as the oxygen level is greater than [*O*_2_]*^c^*, HIF*α* is efficiently degraded by the pathway **p**_7_ and maintains a very low level, and the HIF*α*:ARNT:HRE level is also low (Equation 38). When the oxygen level drops below [*O*_2_]*^c^*, the system switches to the pathway **p**_1_, and HIF*α* stabilizes with a large concentration (*b*_2_/*a* large). This triggers the sharp increase of HIF*α*:ARNT:HRE. The smaller *k*_2_ is, the larger *b*_2_/*a*, and the sharper the HRE occupancy response (see Figure 8). Also, the validity of our analytical approximation is justified by the close resemblance of Figure 8B and 8C.

**Figure 9:**
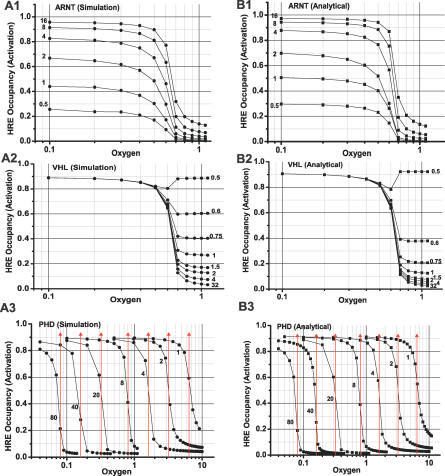
Dependence of the Oxygen Response Curves on the Amounts of Protein Components in the System The red lines in A3 and B3 represent the transition locations calculated from Equation 28.

The three major results of Kohn [1] involve HRE occupancy as a function of the oxygen concentration. The dependence of the curve on ARNT, VHL, and PHD are obtained by both simulation (Figure 9A) and analysis (Figure 9B) and are explained as follows.

**ARNT dependence.** We need only to analyze the pathway **p**_9_. The amount of ARNT does not affect the shape of the response curve or the location of the sharp transition, because **p**_9_ is the pathway for HRE expression, while **p**_7_ is responsible for the sharpness. HIF*α*:ARNT would not be generated without ARNT and there would not be expressions of HRE for any level of oxygen. At high oxygen levels, the concentrations are similar because HRE occupancy is low anyway. At low oxygen levels, low levels of ARNT will give low HIF*α*:ARNT and then low HRE occupancy.

**VHL dependence.** VHL is present in both **p**_7_ and **p**_9_. At high oxygen levels, one should analyze **p**_7_ because it is the major pathway for HIF*α* degradation. The VHL source will affect the upstream HIF*α*. If VHL concentration is low, it cannot degrade HIF*α* fast, and the system yields high HRE occupancy. At low oxygen levels, **p**_1_ is the major pathway for HIF*α* degradation, which does not depend on VHL.

**PHD dependence.** One interesting property relates to the different locations of the transition. Using the criterion identified by the alternative model without reaction *k*_2_, we can calculate the transition locations as shown in Figure 9B3 for different PHD values. The results are the same as in Figure 9A3. As a matter of fact, considering the **p**_9_ pathway only yields a much simpler analytical solution that is also accurate. This further simplification is due to the fact that the expression of HIF*α*OH:ARNT:HRE is always negligible.

The present model of the hypoxia response network is probably an oversimplified one. Nevertheless, it serves as an important starting point, from both theoretical and experimental perspectives, before a more detailed model can be understood. The present model will be gradually expanded and analyzed, with the input of more quantitative data from future experiments. One advantage of EPA is that the method can easily incorporate mechanistic details as soon as they become available [16]. There are various molecular interactions that can be added to the model. For example, it was demonstrated that HIF influences mitochondrial function by inducing pyruvate dehydrogenase kinase 1 (PDK1) to suppress the tricarboxylic acid (TCA) cycle and thus the aerobic respiration. Then the respiration shifts to be anaerobic, whereby the oxygen resource can be preserved to promote cell survival under hypoxic environment [32–34]. Another subject we are interested in is the inclusion of ROS in the network. It has been established that ROS affects HIF*α* degradation through Fe^2+^ [7–10]. The direct hydroxylation of HIF*α* by oxygen requires Fe^2+^. Under hypoxia conditions, however, ROS increases dramatically and consistently removes Fe^2+^ via oxidation to Fe^3+^. Together with the shortage of oxygen, this makes the HIF*α* degradation through the VHL pathway even more difficult. Consequently, the transition would be faster and sharper. Analysis should focus on the explanation of the coexistence of two oxygen sensing components, a matter that does not appear to be settled as yet.

**Figure 10:**
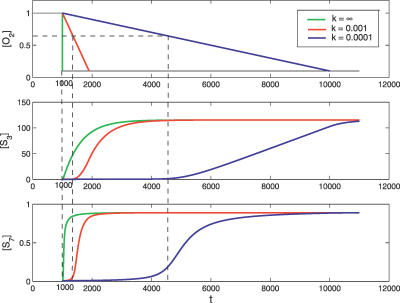
The Responses of [*S*_3_] and [*S*_7_] as the Oxygen Concentration Decreases from 1.0 to 0.1 The oxygen decreases linearly, [*O*_2_] = 1.0 − *k*_t_. The green, red, and blue lines correspond to the cases *k* = ∞ (abrupt decrease), *k* = 0.001, and *k* = 0.0001, respectively. The horizontal dotted line illustrates the critical point [*O*_2_]*^c^*. The vertical dotted lines show that the responses become prominent only after the oxygen concentration decreases below [*O*_2_]*^c^*.

To obtain the dynamical response when the oxgen changes continuously in time, Equations 1–13 (the full model) are integrated. Figure 10 shows the temporal changes of [HIF*α*] and [HIF*α*:ARNT:HRE] as responses to the oxygen decreasing from 1.0 to 0.1 with different rates. One sees that [*S*_3_] and [*S*_7_] increase prominently only after [*O*_2_] decreases below [*O*_2_]*^c^*. The faster [*O*_2_] decreases, the more rapid the responses are. In particular, when [*O*_2_] abruptly jumps from 1.0 to 0.1, the responses ensue promptly. However, it is worth noting that one cannot tell practically how fast the responses are because the time scale is unknown. Indeed, the model is dimensionless and no units are given. Nevertheless, the sharp curves illustrate that the responses are very sensitive to the oxygen concentration and imply that the system can provide a timely response under hypoxia. Physiologists have long been puzzled by the ceaseless HIF*α* cycle, characterized by both abundant generation and efficient degradation, which seems to be a highly wasteful process. Our analysis provides a reasonable explanation. To deal with a sudden environmental change from nomoxia to hypoxia, an organism must respond in time to trigger the genes necessary for adapting to the new environment. To achieve such a sharp response, a high HIF*α* generation potential is necessary. Since the hypoxia conditions are rare, an efficient degradation pathway has to be designed to maintain a low HIF*α* under nomoxia. The HIF*α* cycle is indeed uneconomic, but it appears useful in helping the cell respond to sudden, unpredictable changes in its environment.

In summary, we have obtained an accurate analytical solution to the hypoxia response network and have provided a complete explanation of the switch-like behavior first observed and modeled in [1]. The first step of our analysis applied the EPA technique to a reduced, yet complete system that resulted in exposing ten independent pathways, allowing us to focus on analyzing the pathways relevant to HRE occupancy. The analysis showed that the sharp response of HRE occupancy is due to the switch between the pathway **p**_7_ (**p**_1_) that degrades HIF*α* under nomoxia (hypoxia).

## Materials and Methods

In following the law of mass conservation, a particular reactant through each reaction can be written in the form of homogeneous linear equations,

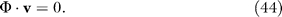



Here **Φ** is an *m* × *m* stoichiometric matrix, where *m* is the number of metabolites and *n* is the number of reactions taking place within the network, with each element

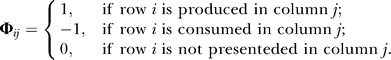


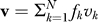
 **,** where *N* = dim Nul**Φ** is the dimension of the null space of **Φ**, *v_k_* is the basis vector that corresponds to the *k*-th pathway, and *f_k_* is the flux through the *k*-th pathway.

The determination of flux *f_k_* requires numerical calculations. Note that *v_k_* does not depend on *f_k_* and can be obtained solely from **Φ**. That is, one can decompose the whole network into *N* elementary pathways without any ODE integration involved. The dimension of the null space of **Φ** follows the simple equation

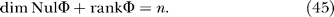



Using Matlab (http://www.mathworks.com), the rank of this 13 × 19 stoichiometric matrix **Φ** is found to be nine, and the dimension of the corresponding null space is thus ten. Also, a set (**B**) of ten independent basis vectors is generated as shown in Figure 3. However, the set is not biologically reasonable due to the negative entries therein. By simple linear transformation, another set (**P**) of ten vectors (Figure 4) is obtained whose entries are all non-negative (either 1 or 0) and are thus biologically feasible.

### Additional results.

The remaining network can be solved analytically with HIF*α*(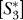 ) already determined. The ODE description is as follows:

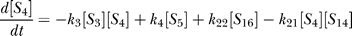


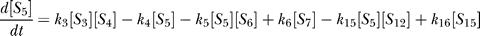


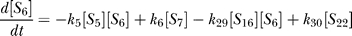


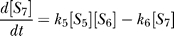


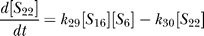


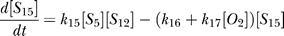


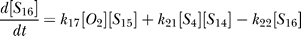


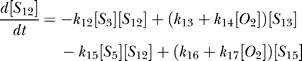


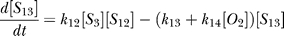



Together with the three constraints of 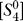 , 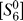 , and 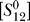 , the steady state equations are expressed as follows:

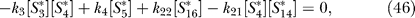


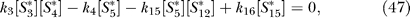


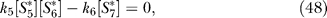


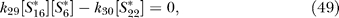


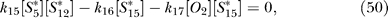


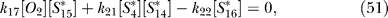


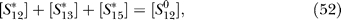


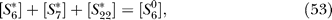


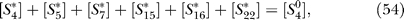
where 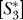 and 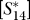 have already been determined from the analysis of **p**_7_. By some reasoning, a quartic equation

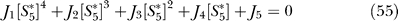
is derived, from which 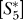 can be obtained. 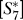 and 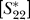 can then be expressed as functions of 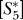 :

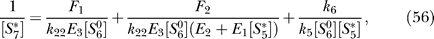


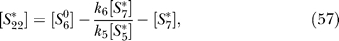
where

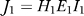


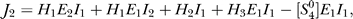


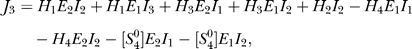


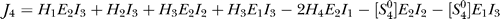


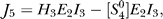


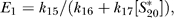


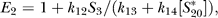


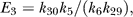


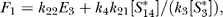


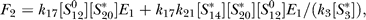


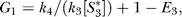


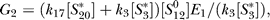


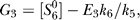


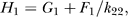


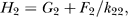


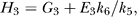


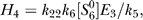


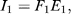


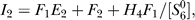


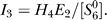



## Supporting Information

### Accession Numbers

Protein accession numbers as listed in Table 1 are from http://www.ncbi.nlm.nih.gov/entrez/query.fcgi?DB=protein.

## Abbreviations

EPA: extreme pathway analysis
HIF: hypoxia-inducible factor
HRE: hpoxia response element
PHD: prolyl hydroxylase
ROS: reactive oxidative species
VHL: von Hippel-Lindau tumor-suppressor protein
